# Supplementary material for: Time‑resolved multi-omic analysis of paclitaxel exposure in human iPSC‑derived sensory neurons unveils mechanisms of chemotherapy‑induced peripheral neuropathy
Source: Cell Death Dis. 2026 Feb 10;17(1):211. doi: 10.1038/s41419-026-08445-2 (PMC12921266; doi:10.1038/s41419-026-08445-2)
Supplement: Supplementary file 15 — Dataset 1. [file 41419_2026_8445_MOESM15_ESM.zip › CDD_Open_Data/04_RNA_Seq/Timeline_panel_plots_etc_2023-01-23_1609.zip!/Panel_Plots_etc/Evidence_Plots/post5d/evidence_plot_standard contrasts_msigdb_reactome_5d_PTX_vs_DMSO_ID6_M630_REACTOME_GLUTAMATE_NEUROTRANSMITTER_RELEASE_CYCLE.pdf]

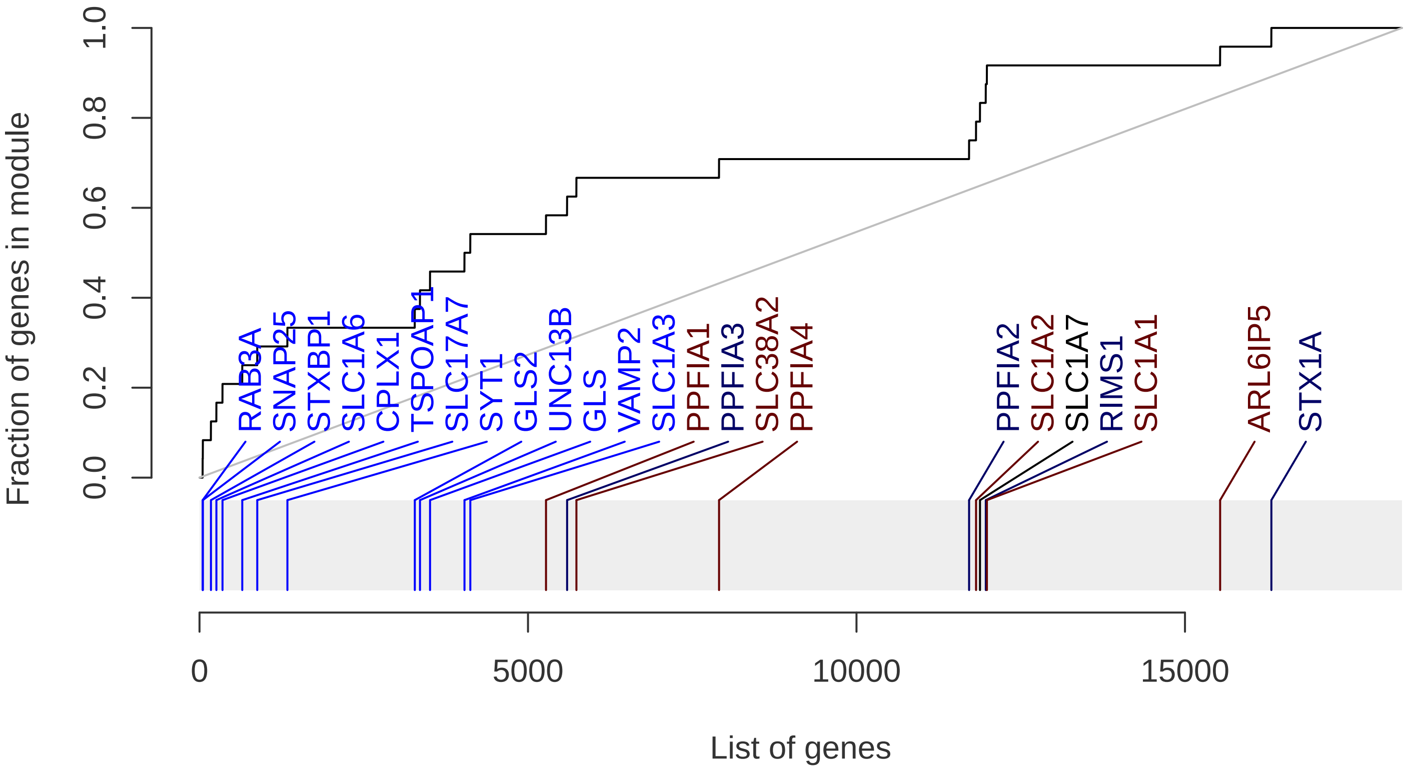

Fraction of genes in module
0.0
0.2
0.4
0.6
0.8
1.0
RAB3A
SNAP25
STXBP1
SLC1A6
CPLX1
TSPOAP1
SLC17A7
SYT1
GLS2
UNC13B
GLS
VAMP2
SLC1A3
PPFIA1
PPFIA3
SLC38A2
PPFIA4
PPFIA2
SLC1A2
SLC1A7
RIMS1
SLC1A1
ARL6IP5
STX1A
0
5000
10000
15000
List of genes
